# Activity-dependent development of synaptic circuits mediates direction selectivity in an axis-specific manner

**DOI:** 10.1016/j.celrep.2025.115897

**Published:** 2025-06-24

**Authors:** Karina Bistrong, Rachana Deven Somaiya, Eugene Y. Liang, Benjamin E. Smith, Marla B. Feller

**Affiliations:** 1 Department of Neuroscience & Helen Wills Neuroscience Institute, University of California, Berkeley, Berkeley, CA 94720, USA; 2 Department of Molecular and Cell Biology, University of California, Berkeley, Berkeley, CA 94720, USA; 3 School of Optometry, University of California, Berkeley, Berkeley, CA 94720, USA; 4 Lead contact

## Abstract

The optokinetic reflex (OKR), which stabilizes images on the retina as a mouse navigates its environment, originates in direction-selective ganglion cells (DSGCs). A mouse model that lacks cholinergic retinal waves, the β2-nAChR-KO mouse, does not develop horizontal direction selectivity but preserves vertical direction selectivity. Here, we demonstrate that the absence of horizontal direction selectivity in β2-nAChR-KO mice results in a loss of the OKR along the horizontal axis, consistent with previous findings on optomotor response. In addition, we observe diminished asymmetric inhibition onto horizontal-preferring DSGCs. In contrast, OKR along the vertical axis and asymmetric inhibition onto vertical-preferring DSGCs is maintained. Dual whole-cell voltage-clamp recordings show that this decrease in asymmetric inhibition is attributable to a reduction in GABAergic conductance between horizontal-preferring DSGCs and their presynaptic partner. These results indicate that, before the onset of vision, spontaneous activity selectively influences the formation of precise wiring essential for motion detection along the horizontal axis.

## In brief

The perturbation of early spontaneous activity differentially impacts horizontal and vertical direction-selective circuits, where horizontal direction selectivity is dramatically reduced. Bistrong et al. explore the behavioral implications and synaptic basis of this loss to reveal the site of activity-dependent plasticity—the maturation of asymmetric wiring in the horizontal direction-selective circuit.

## Graphical Abstract

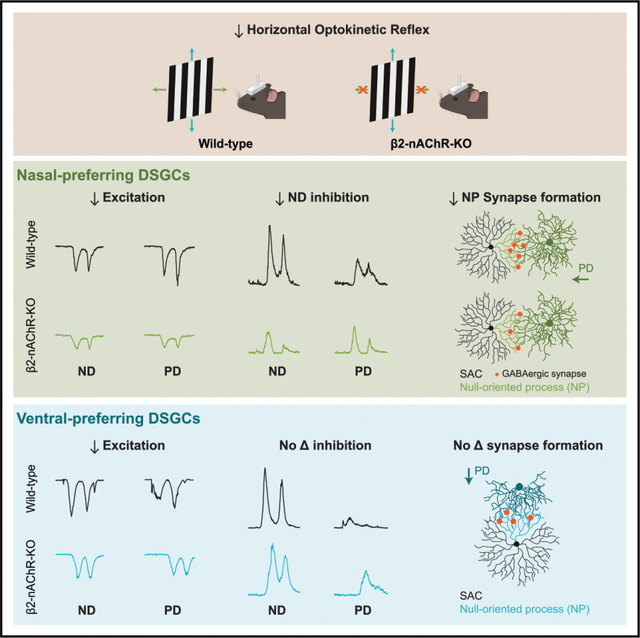

## INTRODUCTION

The mammalian retina contains neural circuits that extract features from the visual scene before transmitting this information to the brain.^1–3^ The role of activity in establishing these circuits remains an open question. In this study, we use the direction-selective circuit as a model to understand how early spontaneous activity affects the development of precise circuit connectivity.

Direction-selective ganglion cells (DSGCs) respond strongly to motion in a preferred direction and weakly to motion in the opposite, or null, direction.^4^ The preferred directions of DSGCs cluster along distinct axes^5–7^ which, in mice, align with the axes defined by optic flow.^8^ DSGCs fall into two broad categories: ON DSGCs, which project to the accessory optic system that mediates gaze stabilization during self-motion,^9,10^ and ON-OFF DSGCs, which project to image-forming brain regions such as the superior colliculus and lateral geniculate nucleus.^11^ The precise synaptic connectivity between DSGC subtypes and their presynaptic partner dictates the preferred direction of a given DSGC.^12^

Direction selectivity emerges early in development,^13,14^ prior to extensive visual experience, in a manner dependent on early spontaneous activity.^7^ During the first postnatal week (post-natal day 0 [P0]–P10), correlated spontaneous activity, referred to as retinal waves, is driven by the volumetric release of acetylcholine (ACh) from starburst amacrine cells (SACs). ACh release activates β2-containing nicotinic ACh receptors (β2-nAChRs) on neighboring SACs and retinal ganglion cells, generating correlated depolarizations that sweep across the surface of the retina.^15^ The β2-nAChR knockout (KO) mouse, which lacks β2 subunit-containing nAChRs, has served as a model for understanding the role of cholinergic retinal waves in the development of several circuits in the visual system.^16,17^ In β2-nAChR-KO retinas, there is a dramatic reduction in direction-selective responses along the horizontal axis, while direction-selective responses along the vertical axis remain intact and tuned.^7^ Consistent with these findings, β2-nAChR-KO mice show optomotor response deficits for horizontal motion but maintain normal optomotor response for vertical motion.^18^

Which elements of the direction-selective circuit are susceptible to this disruption of early spontaneous activity? Direction-selective tuning in DSGCs is influenced by the balance of excitation and inhibition, but the asymmetric response to moving stimuli is largely driven by the asymmetrical release of γ-aminobutyric acid (GABA), with DSGCs experiencing stronger inhibition during null direction motion compared to preferred direction motion.^13,14,19,20^ This asymmetric inhibition is provided by SACs, which release both GABA and ACh. Excitation is mediated by glutamatergic input from bipolar cells and cholinergic input from SACs.^21,22^ Interestingly, direction-selective tuning remains robust over a large range of excitation/inhibition ratios,^23–25^ indicating that asymmetric inhibition has an outsized impact on this computation.

Here, we characterize the optokinetic reflex (OKR) in β2-nAChR-KO mice, which provides a more direct readout of retinal direction-selective tuning than the optomotor response. In addition, we utilize two-photon targeted whole-cell recordings, pharmacology, and conductance modeling to characterize the synaptic basis for the loss of horizontal direction selectivity and the retention of vertical direction selectivity in β2-nAChR-KO retinas. Finally, we employ dual whole-cell recordings to investigate the impact of early spontaneous activity on the synaptic connectivity between DSGCs and SACs. This study offers a comprehensive understanding of the synaptic inputs that drive direction selectivity and suggests a developmental activity-dependent mechanism unique to the synaptic connections between horizontal-preferring DSGCs and SACs that is not present in vertical-preferring DSGCs.

## RESULTS

### β2-nAChR-KO mice have a reduced OKR for horizontal but not vertical motion

Previously, we used population two-photon calcium imaging to show that, in β2-nAChR-KO retinas, horizontal-preferring ON and ON-OFF DSGCs exhibit a symmetric response to visual stimuli, while vertical-preferring DSGCs remain tuned.^7^ In addition, behavioral assessments showed a deficit in horizontal optomotor response in these mice, while vertical optomotor response remains intact.^18^ Here, we revisit these findings by first comparing the spiking patterns of horizontal- and vertical-preferring DSGCs between wild-type and β2-nAChR-KO mice as well as assessing their OKR along the two primary axes in visual space.

**Figure 1.**
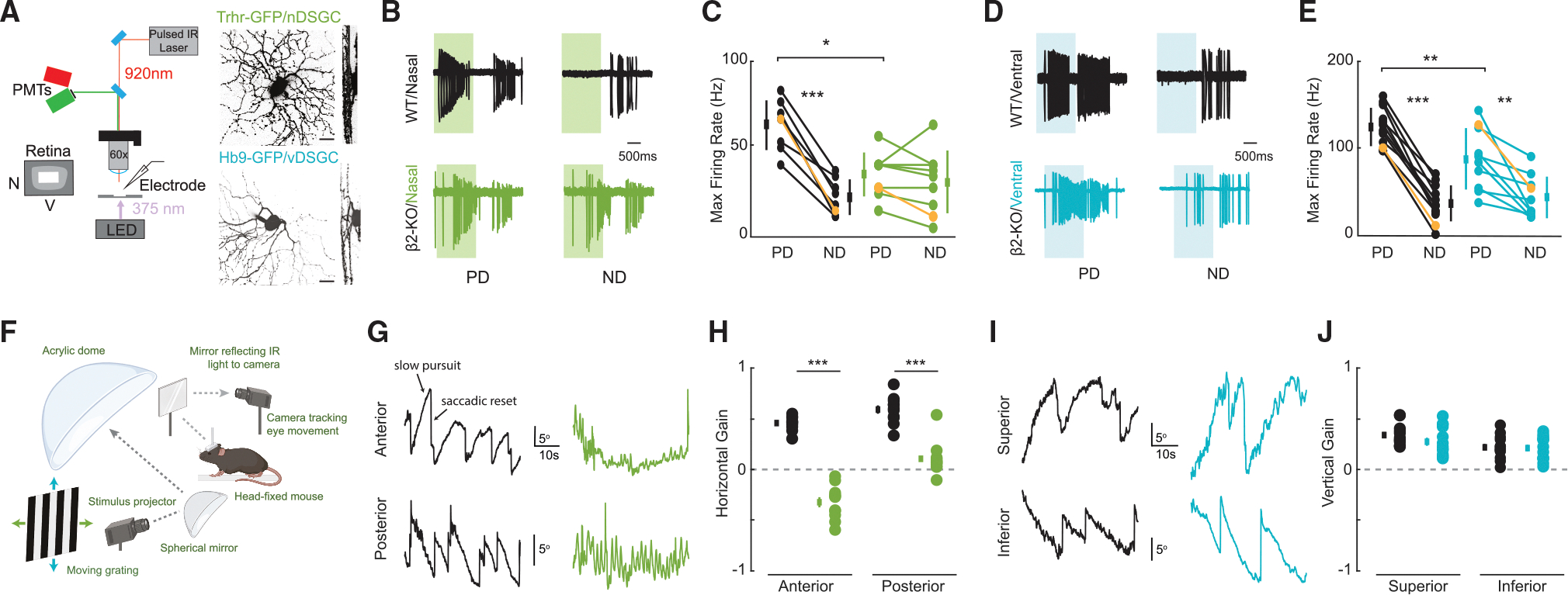
nDSGCs show reduced spike tuning correlated with a disrupted OKR for horizontal but not vertical motion in β2-nAChR-KO mice (A) Schematic showing two-photon microscopy used for targeted whole-cell voltage-clamp recordings (N, nasal; V, ventral). Shown are maximum Z and X-Y projections of example GFP+ nDSGCs (Trhr-GFP) and vDSGC (Hb9-GFP) filled with Alexa 594. Scale bar, 10 μm. (B) Example recorded spike output from example nDSGCs in wild-type (black) and β2-nAChR-KO (green) retinas in the preferred and null directions (PD and ND, respectively). Subsequent analyses were restricted to the highlighted ON response, indicated by the shaded regions. (C) Maximum firing rate for nDSGCs in wild-type (black) and β2-nAChR-KO (green) retinas for the ON response. Yellow lines highlight example cells shown in (A). *n* = 8 nDSGCs across 3 wild-type retinas and 8 nDSGCs across 3 β2-nAChR-KO retinas. *p < 0.05, **p < 0.01, ***p < 0.001; ANOVA followed by Tukey-Kramer post hoc test. Error bars show mean ± standard deviation. (D and E) Same as (B) and (C) but for vDSGCs in wild-type (black) and β2-nAChR-KO (blue) retinas for the ON response. Yellow lines highlight example cells shown in (D). *n* = 12 vDSGCs across 4 wild-type and 10 vDSGCs across 4 β2-nAChR-KO retinas. *p < 0.05, **p < 0.01, ***p < 0.001; ANOVA followed by Tukey-Kramer post hoc test. Error bars show mean ± standard deviation. (F) Schematic of the OKR rig. High-contrast moving visual stimuli are projected onto a hemispherical dome, encompassing the mouse’s entire visual field. The head-fixed mouse is positioned at the center of the rig. Two infrared-sensitive cameras, spaced 12° apart horizontally, capture eye movements and corneal reflections. (G) Representative traces from one wild-type (black) and β2-nAChR-KO (green) mice, illustrating phases of slow pursuit and resetting saccades during posterior and anterior motion. (H) Horizontal gain in response to both posterior and anterior motion for wild-type (black) and β2-nAChR-KO (green) mice. Each dot represents a trial from a mouse, with three mice per genotype, and at least three trials of the same motion presented to each mouse. Positive gain indicates a greater cumulative distance covered during horizontal slow pursuit in the direction of the stimulus, while negative gain reflects a greater cumulative distance in the direction opposite to the stimulus. Note: for anterior motion in β2-nAChR-KO mice, rapid oscillatory eye movements result in a greater cumulative distance traveled in the posterior direction, leading to a negative gain value. ***p < 0.001, unpaired t test. Thick bars represent mean ± SE. (I and J) Same as (G) and (H) but for vertical gain in wild-type (black) and β2-nAChR-KO (blue) mice in response to superior and inferior motion. *n* = 3 wild-type mice and 3 β2-nAChR-KO mice. Thick bars represent mean ± SE. See Figure S1 and Videos S1, S2, S3, S4, S5, S6, S7, and S8 for the OKR to all four cardinal directions.

To compare direction-selective tuning in horizontal- and vertical-preferring DSGCs between wild-type and β2-nAChR-KO mice, we conducted cell-attached recordings during the presentation of a high-contrast moving bar stimulus (8 directions, 45° offset). These recordings were conducted in subpopulations of ON-OFF DSGCs, for which we have transgenic mouse lines, allowing for two-photon targeted recordings: for nasal-preferring ON-OFF DSGCs (nDSGCs, which favor posterior motion in the visual field), we recorded from wild-type and β2-nAChR-KO retinas, where GFP is expressed under either the Trhr or Drd4 promoter^26,27^ (Figure 1A). For ventral-preferring ON-OFF DSGCs (vDSGCs, which favor superior motion in the visual field), we recorded from wild-type and β2-nAChR-KO retinas, where GFP is expressed under the Hb9 promoter.^28^ Consistent with calcium imaging studies,^7^ we observed a reduction in the asymmetry of spiking in the ON pathway of nDSGCs driven by a significant reduction in preferred direction spiking (Figure 1C), while the asymmetry in spiking was maintained in vDSGCs (Figure 1E).

Reduced directional tuning in horizontal- but not vertical-preferring DSGCs is consistent with previous studies indicating that β2-nAChR-KO mice have a reduced ability to track visual stimuli moving along the horizontal axis while retaining the ability to track stimuli along the vertical axis. In this earlier work, visual tracking was assessed using the optomotor response, which involves compensatory head movements that stabilize the visual image during motion.^18^ This response is closely related to the OKR where reflexive eye movements are triggered by global motion in the visual field. Here, we focused on testing differences in the OKR between wild-type and β2-nAChR-KO mice, providing a more direct behavioral assessment of changes in DSGC tuning.^9^

To evoke and measure the OKR, we built a behavioral rig to assess both horizontal and vertical reflexive eye movements in head-fixed animals.^9,29^ The rig displayed full-field moving gratings in the four cardinal directions to mice while an infrared camera tracked the pupil position^30–33^ (Figure 1F). In response to all stimulus directions, all mice exhibited both features of the OKR: cycles consisting of a slow movement (slow pursuit) following the motion direction and a resetting saccade in the opposite direction.

In wild-type mice, we observed a reliable horizontal slow pursuit during horizontal motion and vertical slow pursuit during vertical motion (see example traces in Figures 1G and 1I; see also Videos S1, S2, S3, S4, S5, S6, S7, and S8). Additionally, across all stimulus directions, eye movements exhibited a diagonal offset, indicating minor tracking along the non-dominant axis (Figure S1). In β2-nAChR-KO mice, vertical motion elicited slow tracking movements with frequent resetting saccades, similar to wild-type mice. However, horizontal motion elicited rapid, oscillatory horizontal movements over a small distance, with no tracking and an absence of both key OKR characteristics—sustained slow pursuit and resetting saccades (Figure S1).

To quantify these differences, we extracted the slow pursuit phase of the OKR and measured the total distance the eye traveled during the stimulus presentation. This allowed us to compute the gain, defined as the ratio of eye velocity to stimulus velocity during slow pursuit. Indeed, we observed significant differences in the gain for both posterior and anterior motion (Figure 1H), suggesting an impaired ability to evoke the horizontal OKR in β2-nAChR-KO mice. We observed no significant change in the gain for superior and inferior motion, indicating that a normal vertical OKR is retained in β2-nAChR-KO mice (Figure 1J).

### nDSGCs receive reduced asymmetric inhibition misaligned to the null direction in β2-nAChR-KO retinas

To assess changes in the synaptic inputs to DSGCs in β2-nAChR-KO retinas that underly this dramatic reduction in tuning and motion tracking along the horizontal axis, we performed whole-cell voltage-clamp recordings. We isolated excitatory and inhibitory postsynaptic currents (EPSCs and IPSCs, respectively) during presentation of the same high-contrast moving bar stimulus used to assess spiking (8 directions, 45° offset). ON-OFF DSGCs respond as the bar enters and exits the receptive field, referred to as the ON and OFF response, respectively. We analyzed the ON and OFF responses independently, as they are driven by different pathways.^19,34^

To isolate IPSCs, we held the membrane potential at the reversal potential for cations (0 mV). For each recording, we defined the null direction as the stimulus direction evoking the largest inhibitory current and the preferred direction as the stimulus direction 180° opposite to it. We defined the null direction using the peak IPSC because (1) DSGC tuning is known to be defined by asymmetric inhibition, and (2) there is a small degree of variance when orienting retinas in the recording chamber.

**Figure 2.**
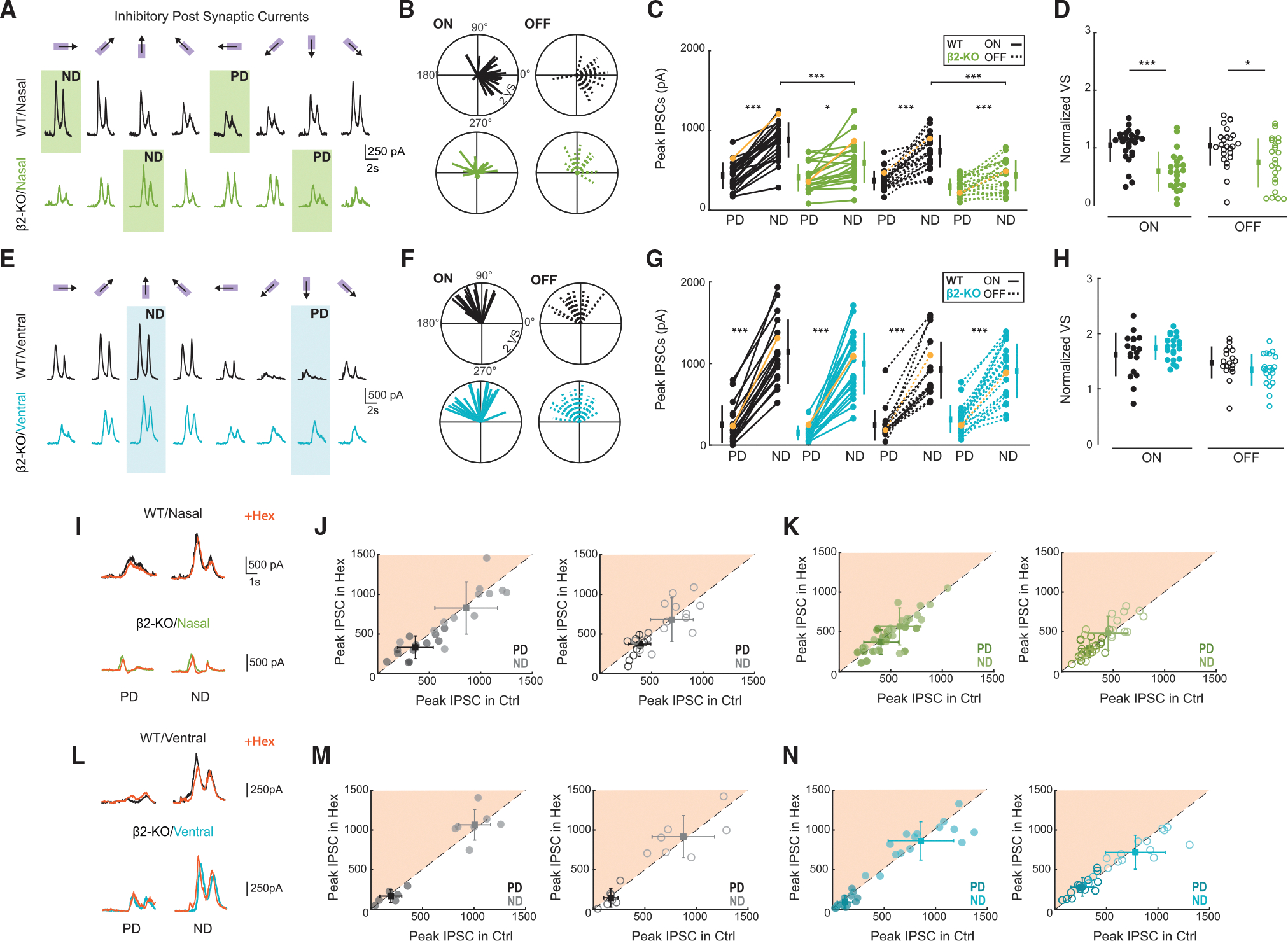
nDSGCs receive reduced asymmetric inhibition that is misaligned in β2-nAChR-KO retinas (A) Average inhibitory postsynaptic currents (IPSCs) recorded from one nDSGC in wild-type (black) and β2-nAChR-KO (green) retinas in response to three repetitions of eight moving bars. The PD and ND of the cell are highlighted. (B) Vector plots of PD of inhibition for wild-type (black) and β2-nAChR-KO (green) retinas for ON (solid) and OFF (dashed) responses. The length of each vector represents the strength of inhibitory tuning (VS, vector sum). 180° is nasal. (C) Peak IPSCs of nDSGCs in response to moving bars in the PD and ND for wild-type (black) and β2-nAChR-KO (green) retinas. The ND of the cell was determined as the direction with the maximum inhibitory response. The PD was determined as 180° from the ND. The ON (solid) and OFF (dashed) components of each response were separated. Yellow lines highlight example cells shown in (A). *n* = 25 nDSGCs across 14 wild-type and 22 nDSGCs across 5 β2-nAChR-KO retinas. *p < 0.05, ***p < 0.001; ANOVA followed by Tukey-Kramer post hoc test. Error bars show mean ± standard deviation. (D) VS normalized to the peak IPSC for all nDSGCs in wild-type and β2-nAChR-KO for ON (filled) and OFF (open). Each data point is a cell. *p < 0.05, ***p < 0.001; unpaired t test. Error bars show mean ± standard deviation. (E–H) Same as (B)–(E) but for vDSGCs in wild-type (black) and β2-nAChR-KO retinas (blue). *n* = 18 vDSGCs across 5 wild-type and 21 vDSGCs across 5 β2-nAChR-KO retinas. ***p < 0.001, ANOVA followed by Tukey-Kramer post hoc test. Error bars show mean ± standard deviation. (I) Example IPSCs from one cell in response to PD and ND moving bars in wild-type (black) and β2-nAChR-KO (green) retinas in control and in the presence of hexamethonium (Hex; orange, 10 μM). (J) Peak IPSCs of nDSGCs in wild-type retinas in control and Hex in the PD (dark) and ND (light) for the ON (left, filled) and OFF (right, open) responses. *n* = 12 nDSGCs across 3 wild-type mice.*p* > 0.05, paired t test. Error bars show mean ± standard deviation. (K) Same as (J) but for IPSCs in of nDSGCs in β2-nAChR-KO (green) retinas. *n* = 19 nDSGCs across 5 β2-nAChR-KO mice. *p* > 0.05, paired t test. Error bars show mean ± standard deviation. (L–N) Same as (I)–(K) but for IPSCs of vDSGCs. *n* = 7 nDSGCs across 2 wild-type mice and 15 cells across 3 β2-nAChR-KO mice. *p* > 0.05, paired t test. Error bars show mean ± standard deviation.

Wild-type nDSGCs had larger IPSCs in the null direction than in the preferred direction for both the ON and OFF response and had IPSCs that were well aligned to the null direction (Figures 2A and 2B), indicating larger asymmetric inhibition tuned to null direction stimuli (0°; Figures 2C and 2D). Interestingly, β2-nAChR-KO nDSGCs had IPSCs that were misaligned from the null direction, showing a bias toward the dorsal direction (90°; Figures 2A and 2B). In addition, the amplitude of IPSCs was significantly reduced for null direction stimulation, regardless of the alignment (Figure 2C), resulting in a significant reduction of tuned inhibition (Figure 2D). This reduction in the amplitude of IPSCs in wild-type and β2-nAChR-KO retinas was unaffected by application of the global nAChR antagonist hexamethonium (Hex; 100 μM; Figures 2I–2K). Hence, the loss of asymmetric inhibition in nDSGCs is not due to the acute loss of nAChR-mediated signaling, implying that it is established via a developmental process driven by early spontaneous activity.

Wild-type vDSGCs had larger IPSCs in the null direction than in the preferred direction, which, in contrast to nDSGCs, persisted in β2-nAChR-KO retinas (Figure 2E). We found no significant differences between wild-type and β2-nAChR-KO in the alignment, amplitude, or tuning of IPSCs in vDSGCs (Figures 2F–2H). Additionally, there were no observed differences in the IPSC amplitude in the presence of Hex (Figures 2L–2N).

Taken together, the axis-specific reduction in asymmetric inhibition in nDSGCs in response to horizontal motion and the 90° shift in null direction alignment indicates a disruption of the normal developmental process that mediates consistent asymmetric wiring of inhibition onto these cells.

### Dual whole-cell recordings reveal that nDSGCs receive reduced GABAergic conductance from null-oriented SAC processes in β2-nAChR-KO retinas

**Figure 3.**
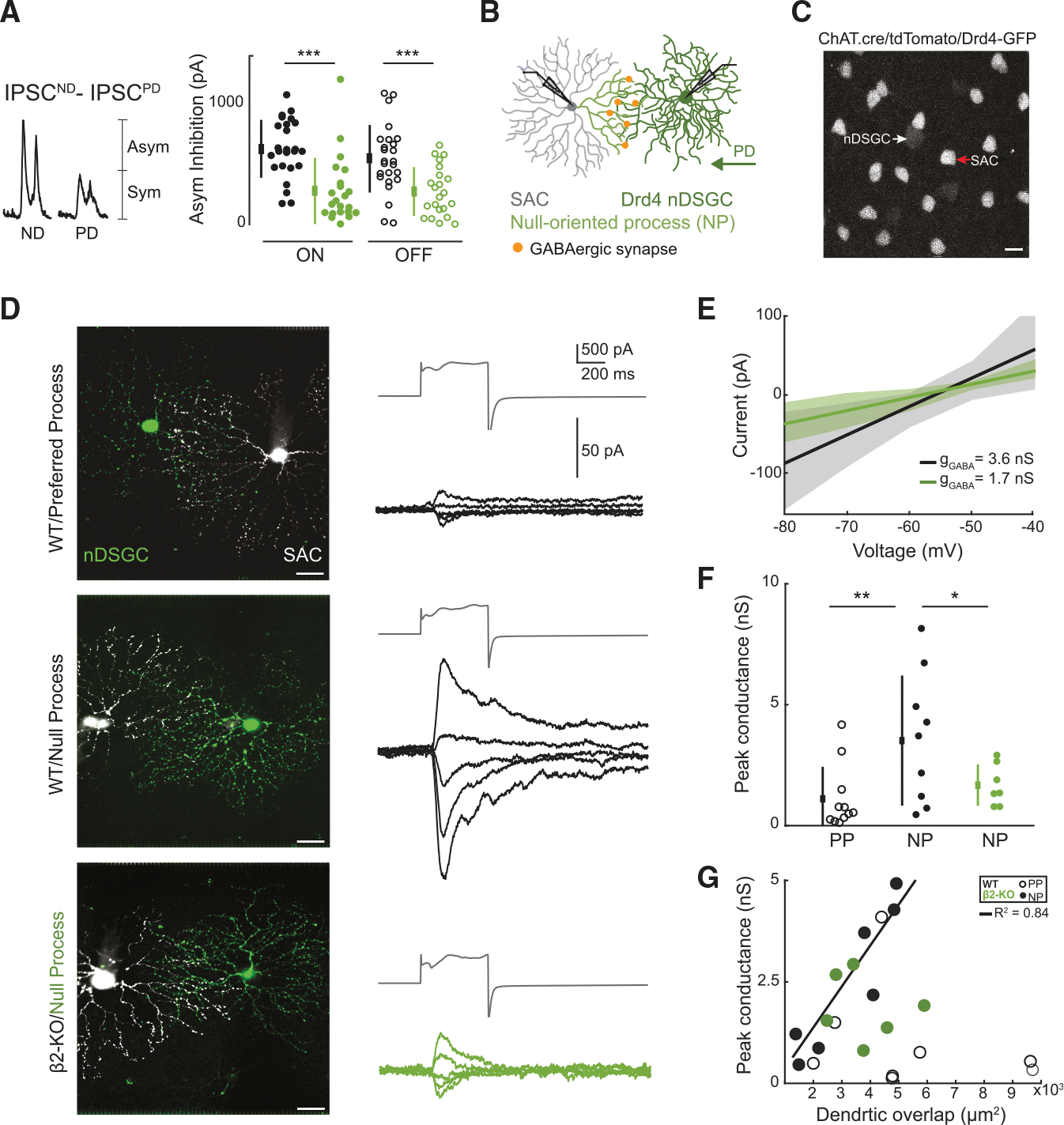
nDSGCss receive reduced GABAergic conductance from null-oriented SAC processes in β2-nAChR-KO retinas (A) Asymmetric inhibition of wild-type (black) and β2-nAChR-KO nDSGCs (green) for both the ON (filled) and OFF (open) responses. The asymmetric inhibitory component was calculated by subtracting the PD IPSC from the ND IPSC. ***p < 0.001, unpaired t test. (B) Schematic depicting asymmetric wiring and dual whole-cell recording between nDSGCs and SACs. (C) Two-photon image acquired using a 920-nm laser, depicting Drd4-GFP (white arrow) and ChAT.cre-tdTomato (red arrow) expression for dual whole-cell targeting. Scale bar, 20 μm. (D) Left: example images from connected nDSGCs (Alexa 488, green) and SACs (Alexa 594, white). Right: recorded GABAergic conductance from the same nDSGCs in wild-type (black) and β2-nAChR-KO retinas (green) in response to SAC depolarization (gray). Scale bar, 20 μm. We isolated the GABAergic conductance by applying a cocktail of synaptic blockers into the bath solution (50 μM L-AP5, 20 μM DNQX, 1 μM TTX, and 10 μM Hex). (E) Average current-voltage relationship across all recorded null-oriented pairs for wild-type (black) and β2-nAChR-KO (green) retinas, where the slope of each line represents the average GABAergic conductance (g_GABA_), and shaded regions represent the standard deviation. (F) Peak GABAergic conductance from preferred- and null-oriented processes (PPs, open circles; NPs, filled circles) in wild-type (black) and null-oriented processes in β2-nAChR-KO retinas (green). *n* = 11 preferred-oriented pairs and 12 null-oriented pairs across 6 wild-type retinas and 7 null-oriented pairs across 4 β2-nAChR-KO retinas. *p < 0.05, **p < 0.01; one-way ANOVA followed by Tukey-Kramer post hoc test. Error bars show mean ± standard deviation. (G) Peak GABAergic conductance as a function of dendritic overlap between nDSGC and SACs. The black line represents correlation for wild-type NPs (black filled circles), where the correlation coefficient (R^2^) equals 0.84. No correlation was found for β2-nAChR-KO NPs (green filled circles) or wild-type PPs (open black circles). Pairs with unclear images were excluded from convex hull analysis. See Figure S2 for dual whole-cell recordings between vDSGCs and SACs.

Asymmetric inhibition onto ON-OFF DSGCs is provided by SACs, where SACs release more GABA during motion in the null direction than in the preferred direction.^12,14^ However, DSGCs also receive “symmetric inhibition,” which we define as equal amounts of inhibition for preferred- and null-direction motion, from other amacrine cells.^35^ Hence, to isolate asymmetric SAC-mediated inhibition, we subtracted the symmetric component (IPSCs generated by preferred-direction stimulation) from IPSCs generated by null direction stimulation. In this analysis, asymmetric inhibition onto nDSGCs in β2-nAChR-KO retinas is significantly reduced (Figure 3A), while asymmetric inhibition onto vDSGCs in β2-nAChR-KO retinas is unaffected (Figure S2A).

To assess whether this reduction in asymmetric inhibition is due to a reduction in asymmetric wiring between nDSGCs and SACs, we performed dual whole-cell voltage-clamp recordings. We restricted our recordings to ON SACs because they provide asymmetric inhibition onto both ON and ON-OFF DSGCs,^36^ and both subtypes present a deficit in tuning to horizontal motion.^7^ In the adult, an individual SAC process forms more GABAergic synapses with the DSGC subtype whose preferred direction is antiparallel to the SAC process (Figure 3B). We recorded the GABAergic conductance from a single nDSGC in response to maximally depolarizing a nearby SAC, situated on either the preferred or null side, where the connected processes are null- or preferred-oriented (NP or PP, respectively; see example pairs in Figure 3D).

In wild-type mice, stimulation of SACs whose null-oriented processes overlap with nDSGC dendrites led to a larger total GABAergic conductance than that observed for preferred-oriented processes (Figures 3E and 3F). There was a large variance in the conductance we observed, which was partially explained by differences in dendritic overlap with nDSGCs and null-oriented SAC processes for a given pair (Figure 3G), as reported previously.^37^ In β2-nAChR-KO retinas, we observed a significant reduction in the GABAergic conductance from null-oriented processes (Figures 3E and 3F). These data indicate that the observed reduction in null direction inhibition onto nDSGCs in β2-nAChR-KO retinas is due to a loss of asymmetric inhibition from SACs. We noted a surprisingly modest conductance between vDSGCs and null-oriented SAC processes in wild-type retinas (Figure S2), which we address in the discussion.

Together, these data indicate a selective activity-dependent reduction of asymmetric inhibition in nDSGCs resulting from a reduction in the formation of GABAergic synapses between nDSGCs and null-oriented SAC processes.

### Both nDSGCs and vDSGCs receive reduced excitation in β2-nAChR-KO retinas

The excitatory component of the direction-selective response is comprised of glutamate and ACh and, as assayed by whole-cell recordings, is mostly symmetric, though there are some reported asymmetries.^38,39^ The cholinergic contribution to direction selectivity is influenced by numerous factors and is highly dependent on stimulus properties, including size, speed, and contrast.^21,22,40^ In the present study, we used high-contrast moving bar visual stimuli, which minimally engages asymmetric cholinergic signaling. Additionally, previous studies did not directly compare the contribution of ACh across the DSGC subpopulations or consider differences between the ON and OFF pathways.

**Figure 4.**
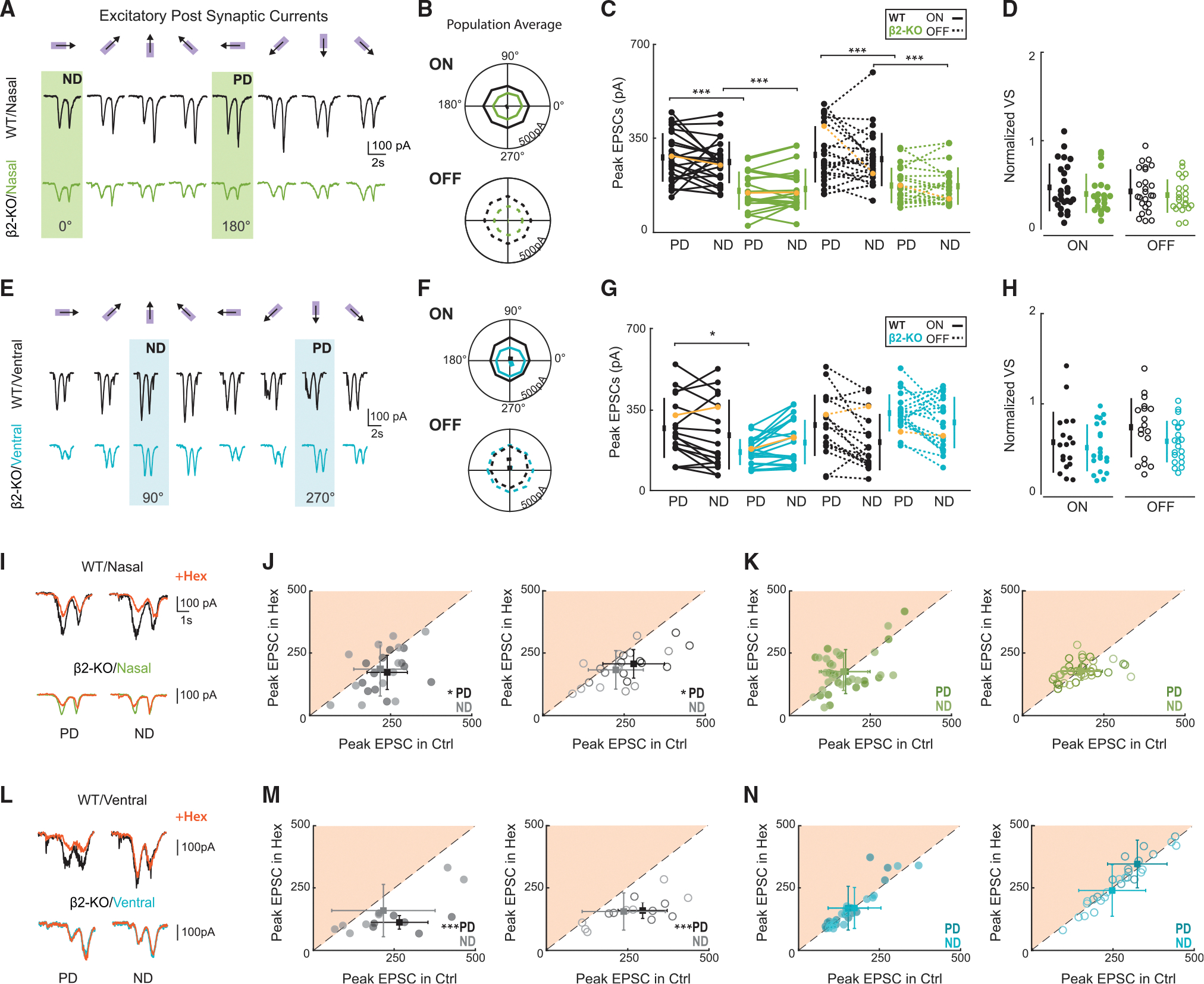
nDSGCss receive reduced excitation in all directions in β2-nAChR-KO retinas due to lack of β2-nAChRs (A) Average excitatory postsynaptic currents (EPSCs) recorded from one nDSGC in wild-type (black) and β2-nAChR-KO (green) retinas in response to three repetitions of eight moving bars. The PD and ND of the cell are highlighted. (B) Average excitatory tuning curves across eight directions for all recorded nDSGCs for ON (solid) and OFF (dashed) in wild-type and β2-nAChR-KO retinas. The line represents the average VS of responses. (C) Peak EPSCs of nDSGCs in response to moving bars in the PD and ND for wild-type (black) and β2-nAChR-KO (green) retinas. The ND and PD of excitation were determined by the ND and PD of inhibition. The ON (solid) and OFF (dashed) components of each response were separated. Yellow lines highlight example cells shown in (A). *n* = 25 nDSGCs across 14 wild-type and 22 nDSGCs across 5 β2-nAChR-KO retinas. ***p < 0.001; ANOVA followed by Tukey-Kramer post hoc test. Error bars show mean ± standard deviation. (D) VS normalized to the peak EPSC for all nDSGCs in wild-type and β2-nAChR-KO for ON (filled) and OFF (open). Each data point is a cell. Error bars show mean ± standard deviation. (E–H) Same as (A)–(D) but for vDSGCs in wild-type (black) and β2-nAChR-KO retinas (blue). *n* = 18 vDSGCs across 5 wild-type and 21 vDSGCs across 5 β2-nAChR-KO retinas. *p < 0.05; ANOVA followed by Tukey-Kramer post hoc test. Error bars show mean ± standard deviation. (I) Example EPSCs from one cell in response to PD and ND moving bars in wild-type (black) and β2-nAChR-KO (green) retinas in control and in the presence of Hex (orange, 10 μM). (J) Peak EPSCs of nDSGCs in wild-type retinas in control and Hex in the PD (dark) and ND (light) for the ON (left, filled) and OFF (right, open) responses. *n* = 12 nDSGCs across 3 wild-type mice. *p < 0.05, paired t test. Error bars show mean ± standard deviation. (K) Same as (J) but for EPSCs in of nDSGCs in β2-nAChR-KO (green) retinas. *n* = 19 nDSGCs across 5 β2-nAChR-KO mice. N.S., paired t test. Error bars show mean ± standard deviation. (L–N) Same as (I)–(K) but for EPSCs of vDSGCs. *n* = 7 nDSGCs across 2 wild-type mice and 15 cells across 3 β2-nAChR-KO mice. ***p < 0.05, paired t test. Error bars show mean ± standard deviation.

To assess how excitatory inputs are altered in β2-nAChR-KO retinas, we isolated EPSCs by holding the membrane potential at the reversal potential for chloride (−60mV) during presentation of the same high-contrast moving bar stimulus. In β2-nAChR-KO nDSGCs, the amplitude of EPSCs was reduced across all stimulus directions, with a significant reduction in the preferred and null directions (Figures 4B and 4C), which did not impact EPSC tuning (Figure 4D). In β2-nAChR-KO vDSGCs, the amplitude of preferred direction EPSCs was significantly reduced (Figures 4F and 4G); however, this reduction did not impact EPSC tuning (Figure 4H). Though a subset of wild-type nDSGCs and vDSGCs had tuned EPSCs, the strength of this tuning was weak and inconsistent across cells (Figures 4D and 4H).

To assess whether the reduction in EPSC amplitude in β2-nAChR-KO retinas is the result of reduction in nAChR-mediated signaling, we compared EPSC tuning in the absence and presence of the global nAChR antagonist Hex (100 μM). Results were highly variable from cell to cell, though there were a few consistent trends. For nDSGCs and vDSGCs, we observed a significant reduction in EPSC amplitude in the presence of Hex in wild-type retinas for preferred direction stimulation, but this difference was not significant for null direction stimulation. These Hex-associated changes were absent in β2-nAChR-KO retinas (Figures 4I–4N).

These data indicate that the decrease in EPSC amplitude can be attributed to a loss of nAChR-mediated signaling, consistent with recent studies implicating a contribution of nAChR to the overall tuning in DSGCs,^21,22^ though the role of nAChRs may be more limited in nDSGCs than vDSGCs. These findings suggest that different DSGC types have different contributions of β2-nAChR-mediated ACh signaling to its excitation and that, even within a given cell, there are differences in the ON and OFF pathway.^34^ Whether these differences are due to differential expression on nAChRs on DSGC ON and OFF dendrites or cholinergic modulation of bipolar cells^41^ remains to be determined.

### Conductance modeling supports that loss of tuning in nDSGCs in β2-nAChR-KO retinas is due to reduced asymmetric inhibition

**Figure 5.**
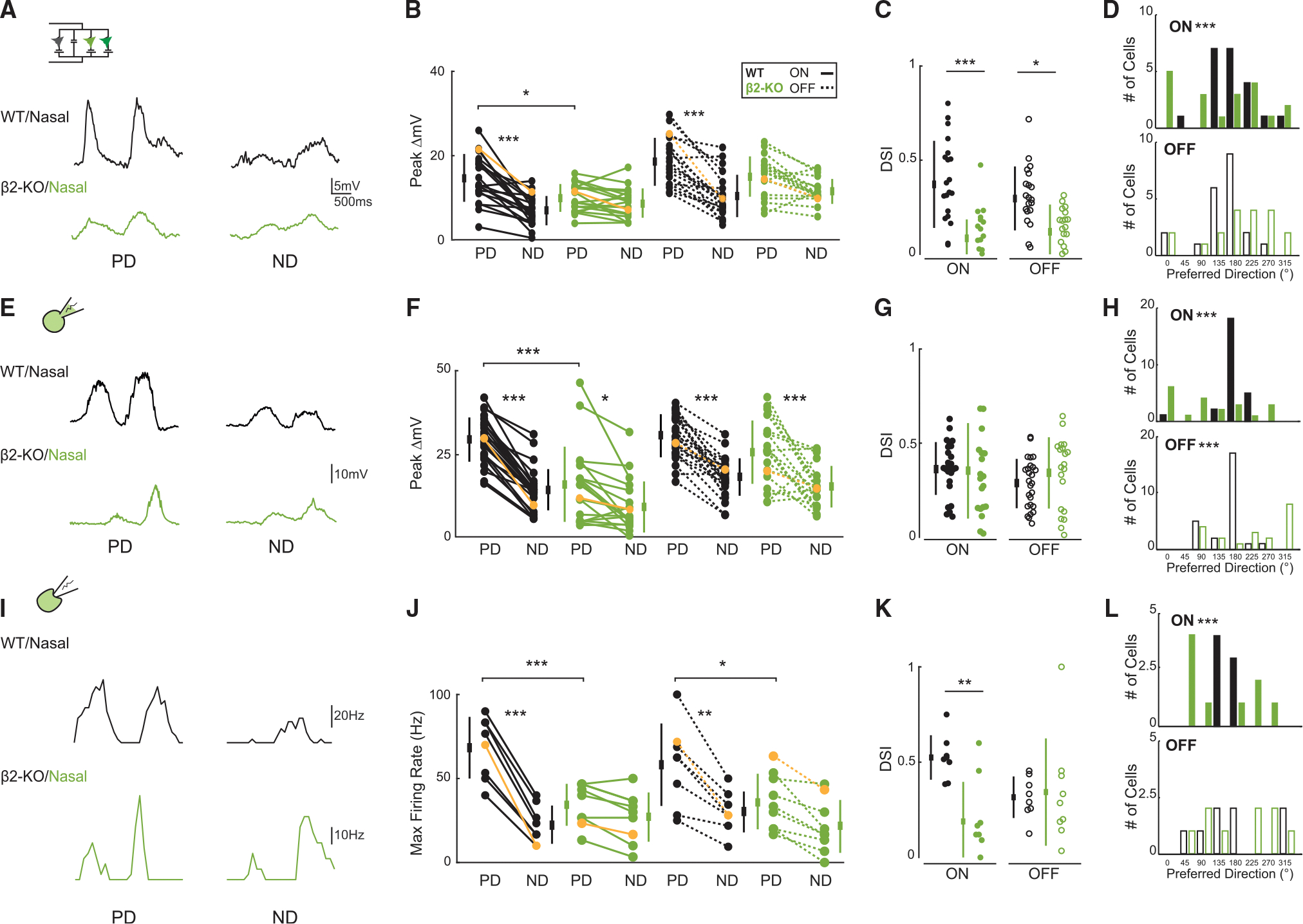
Physiological subthreshold membrane potential and spike recordings are consistent with conductance model predictions for nDSGCss in β2-nAChR-KO retinas (A) Example simulated subthreshold membrane potential from example nDSGC in wild-type (black) and β2-nAChR-KO (green) retinas in the PD and ND. (B) Peak change in simulated subthreshold membrane potential for nDSGCs in wild-type (black) and β2-nAChR-KO (green) retinas for the ON (sold) and OFF (dashed) response. Yellow lines highlight example cells shown in (A). *n* = 25 nDSGCs across 14 wild-type and 22 nDSGCs across 5 β2-nAChR-KO retinas. ***p < 0.001, ANOVA followed by Tukey-Kramer post hoc test. Error bars show mean ± standard deviation. (C) Direction selectivity index (DSI) of simulated subthreshold membrane potential in nDSGCs for ON (filled) and OFF (open) in wild-type (black) and β2-nAChR-KO (green) retinas. *p < 0.05, ***p < 0.001; unpaired t test. Error bars show mean ± standard deviation. (D) Linear histograms representing the PD of simulated subthreshold potential in wild-type (black) and β2-nAChR-KO (green) nDSGCs for the ON (top) and OFF (bottom) responses. Nasal is 180°. ***p < 0.001; Levene’s test for equality of variances. (E) Example recorded subthreshold membrane potential from example nDSGCs in wild-type (black) and β2-nAChR-KO (green) retinas in the PD and ND. Spikes were removed using a low-pass filter or TTX bath application. (F) Peak change in recorded subthreshold membrane potential for nDSGCs in wild-type (black) and β2-nAChR-KO (green) retinas for the ON (sold) and OFF (dashed) response. TTX and “spikes removed” data were grouped together. Yellow lines highlight example cells shown in (E). *n* = 18 nDSGCs across 3 wild-type and 15 nDSGCs across 2 β2-nAChR-KO retinas. ***p < 0.001, ANOVA followed by Tukey-Kramer post hoc test. Error bars show mean ± standard deviation. (G) DSI of recorded subthreshold membrane potential in nDSGCs for ON (filled) and OFF (open) in wild-type (black) and β2-nAChR-KO (green) retinas. *p* > 0.05; unpaired t test. (H) Linear histograms representing the PD of recorded subthreshold potential in wild-type (black) and β2-nAChR-KO (green) nDSGCs for the ON (top) and OFF (bottom) responses. ***p < 0.001; Levene’s test for equality of variances. (I) Average firing rates for example nDSGCs in wild-type (black) and β2-nAChR-KO (green) retinas across 3 trials of PD and ND stimulation. (J) Maximum firing rate for nDSGCs in wild-type (black) and β2-nAChR-KO (green) retinas for the ON (solid) and OFF (dashed) responses. Yellow lines highlight example cells shown in (I). *n* = 8 nDSGCs across 3 wild-type retinas and 8 nDSGCs across 3 β2-nAChR-KO retinas. *p < 0.05, **p < 0.01, ***p < 0.001; ANOVA followed by Tukey-Kramer post hoc test. Error bars show mean ± standard deviation. (K) DSI of maximum firing rate in nDSGCs for ON (filled) and OFF (open) in wild-type (black) and β2-nAChR-KO (green) retinas. ***p < 0.001, unpaired t test. Error bars show mean ± standard deviation. (L) Linear histograms representing the PD of spike output in wild-type (black) and β2-nAChR-KO (green) nDSGCs for the ON (top) and OFF (bottom) responses. ***p < 0.001, Levene’s test for equality of variances. See Figure S3 for the same analysis for vDSGCs.

Above, we described the changes in synaptic inputs onto DSGCs in β2-nAChR-KO retinas. To assess whether these changes in synaptic input are sufficient to explain the observed changes in tuning in nDSGCs but not vDSGCs (Figure 1), we implemented a simple conductance model. First, for each neuron, we integrated our measured excitatory, inhibitory, and resting conductance (see example cells in Figure S3A) to simulate the subthreshold V_m_ induced by preferred and null direction stimulation (V_m_^PD^ and V_m_^ND^, respectively), assessing the impacts on both the ON and OFF pathways. There were no significant differences in the resting conductance for nDSGCs or vDSGCs in wild-type and β2-nAChR-KO retinas (g_Leak_ ≈ 3.3nS). Second, we compared the results of the conductance model with measured V_m_ based on whole-cell current-clamp recordings (Figures 5E–5H). Finally, we compared the subthreshold V_m_ tuning to spiking output, which is known to be superlinear, enhancing the tuning of DSGCs^9,42^ (Figures 5I–5L).

Focusing first on nDSGCs, we simulated V_m_^PD^ and V_m_^ND^ in wild-type retinas, where V_m_ is strongly tuned to the preferred direction for both the ON and the OFF pathways (Figure 5A). In β2-nAChR-KO retinas, the model predicted a reduction in V_m_ tuning in both the ON and OFF pathways (Figure 5C). In the ON pathway, this reduction in tuning was driven by a significant reduction in simulated V_m_^PD^ (Figure 5B), a prediction consistent with the measured V_m_ (Figure 5F) and spiking recordings (Figure 5J). Hence, the reduction in firing for preferred direction stimulation in nDSGCs is consistent with a reduction in excitatory conductance β2-nAChR-KO retinas (Figure 2).

The impact on the OFF pathway was less consistent across approaches. In contrast to the ON pathway, there was no significant decrease in simulated or recorded V_m_^PD^ in β2-nAChR-KO retinas, though there was a significant decrease in preferred direction spiking (Figure 5J). Similar to the ON pathway, we observed no change in V_m_^ND^, indicating a balanced impact of a decrease in excitatory conductance and null direction inhibition on V_m_ (Figures 5F and 5G).

The differences in V_m_ and spiking for the ON and OFF response suggests a differential impact of nAChR signaling between these pathways. Importantly, the preferred directions of nDSGCs that remained tuned across approaches in β2-nAChR-KO retinas were misaligned from the nasal axis, consistent with misalignment of inhibition in voltage-clamp recordings (180°; Figures 5D–5H and 5L). Note that the spike tuning of the OFF response for many nDSGCs recorded in the wild-type was weaker and more variable in amplitude than that for the ON response (Figure 5K), leading to more variance in the preferred direction (Figure 5L). This weaker tuning, however, was also observed in the simulated (Figure 5D) and recorded subthreshold V_m_ (Figure 5H).

We repeated these comparisons for vDSGCs, where asymmetric inhibition is preserved (Figure S3B). For simulated V_m_ in β2-nAChR-KO retinas, there was a significant reduction in V_m_^PD^ in the ON pathway, which led to a significant reduction in V_m_ tuning predicted by the model, consistent with the observed reduction in preferred direction EPSC amplitude (Figures S3C and S3D). While V_m_ remained tuned in the measured depolarizations (Figures S3F–S3I), there was a reduction in tuning in spike recordings (Figure S3G). Finally, the preferred direction of vDSGCs in β2-nAChR-KO retinas remained well aligned to the ventral direction in both the ON and OFF pathways (270°; Figure S3I).

Overall, the conductance model indicates that, in β2-nAChR-KO retinas, the loss of nAChR-mediated excitation reduces the overall strength of the direction-selective depolarization in both nDSGCs and vDSGCs. Though the overall strength of depolarization in the ON pathway was reduced in vDSGCs in β2-nAChR-KO retinas, spiking remained tuned and was sufficient for maintaining the vertical OKR (Figure 1). Together, these data indicate that asymmetric inhibition, rather than the amount of feedforward excitation, is the dominant determinant of the strength of DSGC tuning and preferred direction alignment, critical for accurate motion detection at the cellular and behavioral levels.

## DISCUSSION

Here, we report that early spontaneous activity is critical for the development of asymmetric inhibition selectively along the horizontal axis of the direction-selective circuit in mouse retina. Using a mouse model with disrupted early spontaneous activity, we find that, although both nDSGCs and vDGSCs receive less symmetric excitatory input, only nDSGCs receive reduced asymmetric inhibitory input that is misaligned to the null direction. Dual whole-cell voltage-clamp recordings show that the mechanism underlying this reduced asymmetric inhibition is a reduction in asymmetric connectivity between nDSGCs and null-oriented SAC processes. Based on conductance modeling, these changes in synaptic organization are sufficient to explain the reduction in tuning observed in the horizontal direction-selective circuit and the retention of tuning in the vertical circuit. Overall, our study reveals that different direction-selective circuits within the retina have differential sensitivity to early activity manipulations that manifest at the level of inhibitory synapse formation.

### Potential mechanisms underlying axis-specific activity-dependent formation of GABAergic synapses

Our findings demonstrate that activity during development generated by SACs is essential for establishing asymmetric inhibitory synaptic connections that form the basis of horizontal direction selectivity in the retina. This asymmetry comprises more GABAergic synapses between SAC processes that are antiparallel to the preferred direction of a given DSGC subtype.^12,43^ During development, this precise connectivity is achieved through an increase in synapse number rather than changes in synaptic efficacy,^44^ indicating that activity primarily regulates synapse formation in this circuit. While the molecular mechanisms of activity-dependent changes in glutamatergic synapses have been extensively studied, how activity shapes the development of GABAergic synapses remains far less understood.^45,46^

Several studies have described how activity in β2-nAChR-KO mice is disrupted. Both *in vitro* and *in vivo* studies indicate that, during the first postnatal week, retinal waves in β2-nAChR-KO mice are significantly less frequent and induce weaker depolarizations in retinal ganglion cells.^16,47,48^ Glutamatergic waves normally begin around P10–P11 but appear as early as P8 in β2-nAChR-KO mice.^15^ At P8–P11, wild-type mice show an over-representation of retinal waves originating in the temporal retina and propagating nasally.^49,50^ There is some disagreement about whether glutamatergic waves in the β2-nAChR-KO retain their propagation bias.^49,50^ *In vitro* studies indicate that the propagation bias persists in β2-nAChR-KO mice,^7,16^ while it is reduced *in vivo* to pharmacological manipulations in the retina.^50^ Disrupting propagation bias of both cholinergic and glutamatergic waves *in vivo* decreases the strength of direction-selective tuning in the superior colliculus.^51^ Whether this is the case in the retina remains to be determined.

One mechanism by which an overall reduction in retinal wave activity might disrupt synapse formation in β2-nAChR-KO mice is by reducing GABA release from SACs. Indeed, mice with a mutation in the gene *FRMD7*, a model for human congenital nystagmus,^52,53^ also lack horizontal direction selectivity.^31^ *FRMD7* is expressed exclusively in SACs in the retina, indicating that disruption of presynaptic processes could impact the assembly of direction-selective circuits. Importantly, these studies suggest that different types of disruptions—whether localized to the presynapse, as in *FRMD7* mutants, or dependent on neural activity, as in β2-nAChR-KO mice—can have differential effects on horizontal and vertical direction-selective circuits. In the visual cortex, knockdown of glutamic acid decarboxylase 67, the primary enzyme responsible for GABA synthesis, in interneurons significantly impairs the formation of perisomatic synapses on pyramidal neurons.^54^ In the retina, however, the effects of GABA modulation appear to be more nuanced. While complete blockade of GABA release has been shown to increase the somatic clusters of GABA_A_ receptors on bipolar cell terminals as well as on DSGC somata, selective reduction of GABA release from SACs does not alter the distribution of these receptors on either the dendrites or the somata of DSGCs.^55^ However, such influence across the different DSGC subtypes is less explored.

Another possibility is that loss of excitatory signaling during the first postnatal week influences GABA_A_ receptor synapse formation. For example, in the hippocampus, GABA is depolarizing early in development and activates nascent NMDA receptors (NMDARs), which orchestrate GABAergic synapse assembly. Genetic deletion of functional NMDARs in embryonic development disrupts this process.^56^ However, it remains unclear whether the disruption of GABAergic or excitatory signaling are primarily responsible for altered synapse formation onto DSGCs. Regardless, these findings raise a critical question: what are the molecular mechanisms through which these signaling pathways exert their effects?

Activity-dependent molecular regulation likely occurs in the postsynaptic cell, given that DSGCs with different preferred directions have distinct transcriptomes. A comparison of the transcriptomes of nDSGCs and vDSGCs revealed numerous differentially expressed genes linked to synaptogenesis.^57^ However, despite these substantial differences, horizontal-preferring DSGCs in wild-type and β2-nAChR-KO retinas exhibit only modest transcriptional differences.^58^ This suggests that early spontaneous activity influences GABAergic synaptogenesis primarily at the post-transcriptional or post-translational level. One example of activity-dependent post-transcriptional regulation has been observed in the hippocampus, where activity impacts the local translation of the protein matrix metalloproteinase 9, which is crucial to regulate spine morphology.^59^ Another potential pathway could involve gephyrin, the primary scaffold protein of inhibitory synapses, which interacts with GABA receptors at an approximately 1:1 ratio.^60^ Gephyrin undergoes activity-dependent post-translational modifications, such as palmitoylation and phosphorylation, which are critical for its clustering and interaction with GABA_A_ receptors.^46,61^ Similar modifications on GABA_A_ receptors themselves may regulate their trafficking and stability on nDSGC dendrites.^62^ Future studies could clarify whether local translation or post-translational modifications in gephyrin or GABA_A_ receptors are more susceptible to activity disruption in horizontal-preferring DSGCs, offering insight into the axis-specific role of retinal waves.

An important consideration when exploring these hypotheses is the timing of activity-dependent effects during development. In β2-nAChR-KO mice, retinal waves are reduced during the first postnatal week, yet asymmetric inhibition in ON-OFF DSGCs does not emerge until P10–P12 and a few days earlier in ON DSGCs.^13,14,57^ This delay suggests that retinal waves exert a prolonged influence on circuit development. Similar long-lasting effects have been observed in hippocampal slices, where activity-dependent spine formation leads to functional synapses even after a day.^63^ Whether the impact of activity-dependent mechanisms in horizontal-preferring DSGCs also operates over extended timescales remains to be determined and would require detailed investigation of these processes over longer periods.

### Development of inhibition underlying vertical direction-selective circuits

Distinct mechanisms mediate the development of vertical and horizontal circuits of direction selectivity, with vDSGCs developing tuned inhibition through activity-independent processes. One possibility is that the preferred directions of vDSGCs mature according to a molecular gradient. Ephrin-B2 and its receptors (EphB1, EphB2, and EphB3) are expressed in gradients along the dorsal-ventral axis of the retina and guide topographic map formation of higher visual circuits.^64–66^ Consistent with this idea, the preferred directions of vDSGCs are not always orthogonal to the nasal-temporal axis but, rather, follow along a vertical axis converging toward a singularity in the ventral retina, a pattern similar to that of ephrin gradients.^7,51^ This organization indicates that a ventral bias may develop by following a molecular gradient emerging from this ventral pole.

Another potential mechanism is that both nDSGCs and vDSGCs initially receive vertically biased inhibition, with horizontal tuning later redirected by activity-dependent refinement. In support of this idea, we observe a 90° shift in tuned inhibition in nDSGCs in β2-nAChR-KO retinas, aligning to the vertical axis (Figure 2). This leads to the idea that vertical inhibition may represent a default state where, later in development, early spontaneous activity drives asymmetric synapse formation between DSGCs and SACs specifically for the horizontal circuit.

Last, we found a surprising inconsistency between anatomical connectivity and functional input onto vDSGCs. In wild-type retinas, we observed comparatively weak GABAergic conductance from null-oriented SAC processes onto vDSGCs (Figures S2C and S2D) despite these cells receiving nearly twice as much asymmetric inhibition as nDSGCs (Figure 1). However, electron microscopy reconstructions show an equal number of synapses formed between both nasal- and ventral-preferring DSGCs and SACs.^12^ This discrepency suggests that vDSGCs—particularly the Hb9+ subpopulation—may reinforce tuned inhibition through a SAC-independent mechanism.

The Hb9+ subpopulation of vDSGCs possess structural and functional features that may support their direction selectivity independent of SAC input. First, their dendritic arbors are displaced vertically from the soma, oriented ventrally, correlated with their directional preference^67,68^ but not with their inhibitory tuning.^69^ Second, in the adult retina, Hb9+ vDSGCs are the only DSGC subtype that exhibit homologous gap-junction coupling, which enhances light-driven synaptic signaling by bringing them closer to threshold.^28^ However, while these features may contribute to tuning of Hb9+ vDSGCs, they are unlikely to explain an extra source of asymmetric inhibition. Last, in response to static, vertically oriented bars, vDSGCs receive inhibition from two sources along the vertical axis: direct SAC input and feedforward SAC inhibition driven by vertically tuned glutamatergic excitation.^70^ Whether this asymmetric inhibitory “boost” is the source of vertically biased inhibition underlying vertical direction selectivity remains to be determined.

In summary, our study demonstrates an activity-dependent process that differentially mediates GABAergic synapse formation, discriminating at the level of the postsynaptic cell, between horizontal and vertical direction-selective circuits.

### Limitations of the study

Here, we find that the loss of retinal horizontal direction selectivity in β2-nAChR-KO, first described by Tiriac et al.,^7^ is due to a decrease in asymmetric inhibition onto nDSGCs. These data indicate that activity in the first postnatal week promotes inhibitory synapse formation between SACs and nDSGCs, while inhibitory synapse formation between SACs and vDSGCs is unaffected. This result contrasts earlier studies, where intraocular injections of activity inhibitors did not affect the development of direction selectivity.^14,71^ We attribute this discrepancy to the limited and transient nature of acute pharmacological blockade, which, in our case, could only be applied every 48 h due to concerns over the integrity of the retina. In fact, recent estimates suggest that these intraocular blockades persist for less than 6 h *in vivo.*^50^

Our conclusion that inhibitory synapse formation between SACs and nDSGCs is disrupted in β2-nAChR-KO is limited by the current lack of understanding of the underlying activity-dependent mechanisms. We found that asymmetric inhibition in nDSGCs is not impacted by acute blockade on nAChRs with Hex. However, an important consideration is that the activity-dependent impact on GABAergic synapse formation depends specifically on β2-nAChR-mediated signaling rather than on GABA signaling driven by strong depolarizations of retinal ganglion cells.^72^ For example, the activation of β2-nAChRs strongly elicit Ca^2+^/calmodulin-dependent protein kinase II-dependent pathways in several parts of the brain, where signaling via β2-nAChRs has been implicated in glutamatergic spine formation.^73,74^ β2-nAChRs are also located at GABAergic synapses in the brain, where they modulate GABA signaling during development^75,76^ and in adulthood.^77^ Future experiments that reversibly block DSGC activity independent of nAChR activation, such as through chemogenetic approaches, could help differentiate between these possibilities.

Another limitation is the uncertainty regarding the acute role of nAChRs in direction-selective tuning. As noted under results, the effect of nAChR antagonists was highly variable, both between cells and within individual cells, across preferred/null axes and the ON/OFF responses. Identifying the source of this variance would require additional experiments with more targeted approaches to isolate the impact of nAChR antagonists on bipolar cells, SACs, and DSGCs.^21,34,38,78,79^

## RESOURCE AVAILABILITY

### Lead contact

Requests for further information and resources should be directed and fulfilled by the lead contact, Marla Feller (mfeller@berkeley.edu).

### Materials availability

This study did not generate unique reagents.

### Data and code availability

- All data reported in this paper will be shared by the lead contact upon request.
- All original code has been deposited at https://github.com/FellerLabDS/DSsynapticBasis.git and is publicly available as of the date of publication. DOIs are listed in the key resources table.
- Any additional information required to reanalyze the data reported in this paper is available from the lead contact upon request.

## STAR★METHODS

### EXPERIMENTAL MODEL AND STUDY PARTICIPANT DETAILS

#### Mice

Mice used in this study were aged from postnatal day 20 to 60 and were of both sexes and were housed in 12-h day/night cycle rooms. To use two-photon to target a subset of nDSGCs, we used Trhr-GFP^27^ and Drd4-GFP mice.^26^ To target a subset of vDSGCs, we used Hb9-GFP^69^ mice. To study the role of spontaneous activity, we used β2-nAChR-KO mice^15^ and bred these with either Hb9-GFP or Trhr-GFP or Drd4-GFP transgenic mice. To target both DSGCs and SACs, we bred β2-nAChR-KO of each GFP+ line with SAC-specific *Chat-IRES-Cre* mice, and floxed *tdTomato* mice originally acquired from the Jackson Laboratory. All animal procedures were approved by the University of California, Berkeley Institutional Animal Care and Use Committee and conformed to the National Institutes of Health *Guide for the Care and Use of Laboratory Animals*, the Public Health Service Policy, and the Society for Neuroscience Policy on the Use of Animals in Neuroscience Research.

### METHOD DETAILS

#### Retina preparation

Mice were anesthetized with isoflurane inhalation and euthanized by decapitation. Eyes were immediately enucleated, and retinas were dissected in oxygenated (95%O2/5%CO2) Ames’ media (Sigma) at room temperature under infrared illumination. Cuts along the choroid fissure were made prior to isolating the retina from the retinal pigmented epithelium. These cuts were made to precisely orient retinas to reduce orientation variability between preparations. Retinas were cut, separating the dorsal and ventral halves to allow flattening, and mounted over a 1- to 2mm^2^ hole in nitrocellulose filter paper (Millipore) with the photoreceptor layer side down. Retinas were stored in oxygenated Ames’ media until use (max 10h). All experiments were performed on retinas in which dorsal-ventral orientation was tracked.

#### Two-photon targeted electrophysiological recordings

Here, we followed the protocol details in.^80^ Oriented retinas were placed under the microscope in oxygenated Ames’ media at 32°C–34°C. GFP+ positive cells were identified using a two-photon microscope tuned to 920nm to minimize bleaching of photoreceptors. The inner limiting membrane above the targeted cell was dissected using a glass electrode. Cell-attached recordings were performed with a new glass electrode (4–5MΩ) filled with Ames’ media. Recordings were performed with the same pipette after obtaining a giga ohm (1GΩ) seal. Current-clamp recordings were performed with a new glass electrode (4–5MΩ) filled with internal solution containing the following, in mM: 115 K+ gluconate, 9.7 KCl, 1 MgCl2, 0.5 CaCl2, 1.5 EGTA, 10 HEPES, 4 ATP-Mg2, 0.5 GTP-Na3, 0.025 Alexa 594 (pH = 7.2 with KOH, osmolarity = 290). Voltage-clamp clamp recordings were performed with a new glass electrode (4–5MΩ) filled with internal solution containing the following, in mM: 110 CsMeSO4, 2.8 NaCl, 20 HEPES, 4 EGTA, 5 TEA-Cl, 4 Mg-ATP, 0.3 Na3GTP, 10 Na2phosphocreatine, and 5 QX-Cl (pH 7.2 with CsOH, osmolarity = 290). Whole-cell recordings were performed with the same pipette after obtaining a giga ohm (1GΩ) seal and breaking into the cell membrane. Holding voltages for measuring excitation and inhibition after correction for the liquid junction potential (−12mV) were −60mV and 0mV, respectively. Signals were acquired using Clampex 10.7 recording software and a Multiclamp 700B amplifier (Molecular Devices), sampled at 10kHz, and low pass filtered at 1 kHz.

#### Two-photon targeted dual whole-cell voltage-clamp recordings

Oriented retinas were placed under the microscope in oxygenated artificial cerebral spinal fluid containing the following, in mM: 119 NaCl, 26.2 NaHCO_3_, 11 D-glucose, 2.5 KCl, 1 K_2_HPO_4_, 2.5 CaCl_2_, and 1.3 MgCl_2_ or Ames’ media supplemented with 2.5 CaCl_2_. The following excitatory synaptic blockers were added, in μM: 50 AP-5, 20 DNQX, and 10 Hexamethonium, and 50 TTX (Tocris Bioscience). Voltage-clamp recordings were performed with a new glass electrode (4–5MΩ) filled with internal solution containing the following, in mM: 110 CsMeSO4, 2.8 NaCl, 20 HEPES, 4 EGTA (0.1 for SACs), 5 TEA-Cl, 4 Mg-ATP, 0.3 Na3GTP, 10 Na2phosphocreatine (for DSGCs) (pH 7.2 with CsOH, osmolarity = 290). Whole-cell recordings were performed with the same pipette after obtaining a giga ohm (1GΩ) seal and breaking into the cell membrane. To test connectivity, SACs were depolarized to 0mv while DSGCs were held at different membrane potentials (−80mV to −40mV), this was repeated 3 times. Holding voltages were corrected for the liquid junction potential (−10mV). Signals were acquired using Clampex 10.7 recording software and a Multiclamp 700B amplifier (Molecular Devices), sampled at 10kHz, and low pass filtered at 1 kHz. To measure dendritic overlap, DSGC and SAC recording pipettes were filled with Alexa Dye 488 and 647, respectively. After recording synaptic currents, two-photon 512 × 512 image stacks of the dye filled cells were acquired with 810nm excitation, using a 1μm step size.

#### Pharmacology

For experiments conducted in Hexamethonium (Millipore Sigma), we diluted Hex to 1μM in Ames’ media, and allowed it to perfuse for 5–10 min at a perfusion rate of 2 mL/min.

#### Visual stimulation for electrophysiology

Visual stimuli were generated via custom MATLAB functions written with Psychophysics Toolbox on a computer running a 60 Hz DMD projector (EKB Technologies) with a 375 nm LED light source. The DMD image was projected through a condenser lens and aligned on each experimental day to the photoreceptor layer of the sample. All stimulus protocols were centered on the soma of the recorded cell and were presented after at least 10 s of adaptation on a dark background (9.4 × 103 R*/rod/s). Both bars and flashing spots were of positive contrast, and equal intensity (2.6 × 105 R*/rod/s). On and Off responses were first assessed with 3 repetitions of a flashing spot (50μm radius). Direction selectivity was assessed with 500μm long and 100μm wide bars moving at 500μm/s. Responses were recorded for at least 3 repetitions of bars moving in 8 block-shuffled directions, each separated by 45°. Each presentation lasted for 4.5 and was followed by 3s interstimulus interval of black background.

### QUANTIFICATION AND STATISTICAL ANALYSIS

#### DSGC light responses

For voltage clamp DSGC recordings during moving bar stimuli, traces were first averaged across the three trials for each direction and inspected to ensure the consistency of the responses across trials. Average traces were baseline subtracted based on the first 500ms of recording or a user defined interval after manual inspection. Peak currents were calculated from average baseline subtracted traces. They were defined as the maximal (IPSC) or minimal (EPSC) points during two separate 1.5 s windows in which the ON and OFF responses occurred. All analysis was computed independently between ON and OFF pathways. This is because the ON and OFF dendrites receive inputs from ON and OFF SACs, respectively, as well as different populations of bipolar cells and, therefore, are driven by different circuits.

For voltage clamp, the preferred direction (PD) of each cell was determined by computing the vector sum based on IPSC responses to 8 stimulus directions. The vector sum was defined as the length and angle of the sum of eight vectors (V_D_) normalized by the peak IPSC response ($r_{\max}$). The magnitude of vector sum reflects the tuning strength (VS), and the angle of vector indicates the direction which elicited the largest inhibitory response. The preferred direction of *the cell* was then determined as the direction 180° away from the angle of the vector.


$$\sum^{n=8}\left(\frac{V_{D}}{r_{max}}\right)$$

For cell attached DSGC recordings during moving bar stimuli, maximum firing rate was calculated by bandpass filtering traces (0.08–2 kHz) and manually identifying a threshold value for spikes on the filtered traces. ON and OFF responses were defined as spikes occurring during two separate 1.5 s windows in which the ON and OFF responses occurred. A sliding window was used to find the maximum firing rate for each of the responses. The average maximum firing rate across the 3 trials were used to calculate the vector sum of the spike responses. Preferred directions for both ON and OFF responses were defined as the angle of the vector sum of maximum firing rate. This was done independently for the ON and OFF pathways.

For current clamp DSGC recordings during moving bar stimuli, traces were first averaged across the trials for each direction. Average responses were baseline subtracted based on the first 500ms of recording. Spikes were removed either by lowpass filtering or with the application of 1mM TTX (Tocris Biosciences). Peak subthreshold potentials were detected for both the ON and OFF responses during two separate 1.5 s windows in which the ON and OFF responses occurred. The peak potential across the 3 trials were used to calculate the vector sum of the subthreshold potential. Preferred directions for both ON and OFF responses were defined as the angle of the vector sum of the subthreshold potential. This was done independently for the ON and OFF pathways.

The direction selectivity index (DSI) was calculated as follows where PD is the preferred direction determined by the vector sum and ND is the direction 180° away from PD:

$$DSI=\frac{PD\;-\;ND}{PD\;+\;ND}$$

#### Dual whole-cell recordings

For dual voltage clamp recordings, traces were first averages across the three trials for each stimulation repetition. The peak current for each holding potential was used to generate an IV curve. A line was fit to the IV data. The slope and intercept of the line was used to calculate the inhibitory conductance and the reversal potential. We controlled for quality of a recording by requiring an R^2^ value for the linear fit of the IV data above 0.80.

#### Convex hull analysis

Convex hull analysis was completed in Simple Neurite Tracer in FIJI using the Convex Hull function. In short, dendritic arbors were outlined using the tracing tool and automatically converted to a convex hull. The convex hull for each DSGC and SAC pair were overlayed, and the overlapping area was measured.

#### Conductance modeling

The contributions of synaptic conductance to tuned depolarizations were simulated via a parallel conductance model implemented in MATLAB. Conductance $G_{\text{Exc}}$ and $G_{\text{Inh}}$ used as model inputs were taken from individual whole-cell voltage clamp recordings response to moving bars and were rectified and trial averaged for each repetition of visual stimulus. For each cell type, a simulated cell’s voltage time series trace was numerically integrated via the forward Euler method:

$$V(t+\Delta t)=V(t)+\frac{dV}{dt}\;*\;\Delta t$$

Where dV/dt was derived from the current flow across an RC circuit with empirically determined values for capacitance $C_{m}$ (80 pF), resting conductance $G_{\text{Leak}}$ (3.3 nS) and resting membrane potential $E_{\text{Leak}}$ (−55 mV):

$$\frac{dV}{dt}=\left[G_{Exc}(t)\;*\;\left(V(t)-E_{Exc}\right)+G_{Inh}(t)\;*\;\left(V(t)-E_{Inh}\right)+G_{\mathrm{Leak}}\left(V(t)-E_{\mathrm{Leak}}\right)\right]/C_{m}$$

The amplitude of simulated depolarizations was compared between preferred and null directions, and direction selectivity indices were calculated for each condition across cell types.

#### Optokinetic reflex characterization

To accurately characterize the OKR in mice, we designed and built a custom behavior rig that presented a full-field, binocular stimulus to behaving mice (https://github.com/Llamero/Optokintetic_Relflex_Rig). The rig was a derivative of Harris & Dunn, 2023.^9^ Specifically, an acrylic hemisphere (⌀ 24 inches, California Quality Plastics) was covered with custom paint that had 50% diffuse reflectivity between 350 and 750 nm (kindly provided by Todd Pringle at Twilight Labs) to limit reflections within the hemisphere. This paint was also designed to not have any photoluminescence excited by nUV or visible wavelengths. Stimulus movies were projected using a liquid light guide coupled DLP (EKB Technologies) that was illuminated using a 20W 385 nm and a 7W 530 nm LED (Luminus Devices). The stimulus was then reflected off a silver-plated machined brass hemisphere (⌀ 6 inches, Wagner) and onto the acrylic dome. To account for distortions during visual stimulus projection, a manually fitted spherical morph was applied using Meshmapper (https://www.paulbourke.net).

To track the pupil accurately, two cameras (Genie Nano M1280-nIR, Teledyne) are focused on the mouse eye. These cameras are placed precisely 12° apart relative to the eye so that the translation of the pupil in the camera plane can then be converted to angular rotation of the eye. Furthermore, there are three 850 nm LEDs illuminating the eye, whose specular reflect off the cornea also serve as fiducials for tracking the relative motion of the mouse eye. A third wide field camera was focused on the dome (Genie Nano M1280, Teledyne), and was synced via a master trigger to the nIR cameras, such that all three cameras recorded synchronously allowing the eye motion in the nIR cameras to be temporally correlated to the motion of the visual stimulus.

#### Visual stimulation for OKR behavior

Visual stimuli were generated using PsychoPy, as described previously.^9,81^ The stimuli consisted of sinusoidal gratings moving in the four cardinal directions, corresponding to 0° (nasal/anterior motion), 90° (superior motion), 180° (temporal/posterior motion), and 270° (inferior motion). Each unidirectional grating was presented for 45 s, preceded, and followed by 5 s of a static grating. Gratings were displayed at a spatial frequency of 0.15 cycles/°, a contrast of 1.0, and a velocity of 2°/s. The presentation order was randomized for each animal. JSON files were generated to store replicate-specific parameters for analysis and each stimulus was presented at least three times per animal.

#### Eye tracking

All experiments were conducted in darkness to minimize light contamination. Mice at least 3 months old underwent stereotaxic surgery for the implantation of a custom-made head post. Following surgery, they were given seven days to recover and were kept in a reverse 12–12 light cycle. Experiments were conducted on animals aged 5 to 10 months. Mice were gradually habituated to the behavioral rig over four days, spending a minimum of 15 min per day in the setup and given chocolate milk intermittently. During this period, static horizontal and vertical gratings were presented for equal durations. For eye-tracking experiments, mice were positioned at the center of the hemisphere. Eye movements were recorded using two infrared cameras, while a third camera captured the mouse and the interior of the hemisphere, where visual stimuli were projected. All recordings were acquired at 100 frames per second.

#### Quantification of OKR

After completing eye-tracking experiments, DeepLabCut was used to train a neural network to label pupil boundaries with eight markers and identify three corneal reflections per frame. This process generated two-dimensional (x, y) coordinates for all markers, which were then used for further analysis. The marker coordinates, including pupil border and corneal reflections, were converted from pixel coordinates to angular positions as described below.

Pupil centers were determined using the Coope method. Briefly, DeepLabCut markers were filtered based on their likelihood values to retain only high-confidence points. These markers were then used to fit a best-fit circle, minimizing residual errors. Frames with fewer than three high-confidence markers (the minimum needed to define a circle) were excluded, and their pupil positions were replaced with the last known high-confidence position. The best-fit circle determined the pupil center in each frame, which was then used to track eye position over time.

Corneal reflection markers, two as horizontal references and one as vertical reference, provided a consistent reference point across replicates and animals. Each of the infrared cameras used to record the animal’s eye movements is aligned with an infrared laser that denotes the surface point normal to the respective camera. Together, these three lasers generated a unique reference point on the cornea, enabling consistent pupil position adjustments across replicates and animals.

Angular position calibration was performed by comparing the average distance between pupil centers in both infrared cameras with the known 12° angular separation between them. This allowed for the derivation of a scale factor to convert pixel coordinates into angular positions. The pupil center positions were adjusted relative to the corneal reflection reference point, and the change in pupil center position between the two cameras was used to calculate the scale factor. Calibration was repeated for each stimulus presentation and animal to account for variations in head position or orientation.

#### OKR analysis and quantifications

Recordings were selected based on which camera provided more high-confidence frames during stimulus presentation. Slow pursuit and saccadic/fast movements were distinguished using a custom Python script. Briefly, cumulative slow pursuit displacement was determined from the angular eye position across replicates. Low-confidence frames were removed by replacing pupil center coordinates with the last known high-confidence values. High-frequency noise was reduced using a Gaussian filter. Eye velocity was calculated as the gradient of the pupil center’s angular position. Frames were classified as saccades or fast movements if the total pupil velocity exceeded 30°/s. After filtering out low-confidence and saccadic frames, cumulative slow pursuit displacement was calculated by integrating displacement over time.

For horizontal gain calculation, we analyze slow pursuit eye movement along the x-axis (both anterior and posterior motion), while vertical gain is calculated based on slow-phase movements along the y-axis (both superior and inferior motion). To accurately reflect tracking behavior, directional signs were assigned to slow-phase movements. Positive sign indicates slow pursuit in the same direction as the stimulus and negative sign indicates slow pursuit movement in the opposite direction of the stimulus. The cumulative distance of slow pursuit is calculated by summing these signed displacements. Horizontal and vertical gain were then computed by comparing cumulative slow pursuit displacement to total visual stimulus displacement.

#### Statistical tests

Details of statistical tests, number of replicates, and *p* values are indicated in the figures and figure captions; *p* < 0.05 was considered significant.

## Supplementary Material

1

2

3

4

5

6

7

8

9

SUPPLEMENTAL INFORMATION

Supplemental information can be found online at https://doi.org/10.1016/j.celrep.2025.115897.

## Figures and Tables

**KEY RESOURCES TABLE:** 

REAGENT or RESOURCE	SOURCE	IDENTIFIER

Chemicals, peptides, and recombinant proteins		

Ames’ Media	Sigma	Cat# A1420-10X1L
Hexamethonium Bromide	Sigma	Cat# 501806478
DL-AP5	Tocris Biosciences	Cat# 010510
DNQX	Tocris Biosciences	Cat# 018910
Tetrodotoxin citrate	Tocris Biosciences	Cat# 1069
Alexa Fluor 594 Hydrazide	Molecular Probes	Cat# A10442
Alexa Fluor 488 Hydrazide	Molecular Probes	Cat # A10436

Experimental models: Organisms/strains		

Mouse: B2-nAChR-KO	A. Beaudet, Baylor University, Waco, TX.	N/A
Mouse: B6.129S6-ChATtm1(cre)lowl/J	The Jackson Laboratory	018957
Mouse: B6; FVB-Tg(Trhr-EGFP)HU193Gsat/Mmucd	Mutant Mouse Regional Resource Center	030036
Mouse: Tg(Drd4-EGFP)W18Gsat/Mmnc	Mutant Mouse Regional Resource Center	000231
Mouse: B2-nAChR-KO/Drd4-GFP	Bred in lab	N/A
Mouse: B2-nAChR-KO/Chat.cre/tdTomato/Drd4-GFP	Bred in lab	N/A
Mouse: Hb9-GFP	The Jackson Laboratory	005029
Mouse: B2-nAChR-KO/Hb9-GFP	Bred in lab	N/A
Mouse: B2-nAChR-KO/Chat.cre/tdTomato/Hb9-GFP	Bred in lab	N/A

Software and algorithms		

MATLAB	MathWorks	https://www.mathworks.com/products/matlab.html; RRID: SCR_001622
ScanImage	Vidrio Technologies	http://scanimage.vidriotechnologies.com/display/SIH/ScanImage+Home; RRID: SCR_014307
FIJI	NIH	https://imagej.nih.gov/ij; RRID:SCR_003070
Simple Neurite Tracer FIJI Plugin	NIH	https://imagej.net/SNT
Custom-made analysis code	This paper	https://github.com/FellerLabDS/DSsynapticBasis.git https://doi.org/10.5281/zenodo.15467959
Clampex 10.7	Molecular Devices	N/A
Psychtoolbox 3.0	Open Source	http://psychtoolbox.org/

## Highlights

- Horizontal optokinetic reflex is dramatically reduced in β2-nAChR-KO mice
- Nasal-preferring DSGCs receive reduced asymmetric inhibition in β2-nAChR-KO mice
- Horizontal asymmetric DSGC-SAC wiring is disrupted in β2-nAChR-KO mice
- Vertical direction selectivity is unaffected by early activity manipulations
