# Supplementary material for: Activity-dependent development of synaptic circuits mediates direction selectivity in an axis-specific manner
Source: Cell Rep. Author manuscript; Available in PMC 2025 Aug 14. (PMC12352496; doi:10.1016/j.celrep.2025.115897)
Supplement: 1 [file NIHMS2099506-supplement-1.pdf]

Cell Reports, Volume *44*

Supplemental information

Activity-dependent development of synaptic circuits mediates direction selectivity in an axis-specific manner

Karina Bistrong, Rachana Deven Somaiya, Eugene Y. Liang, Benjamin E. Smith, and Marla B. Feller

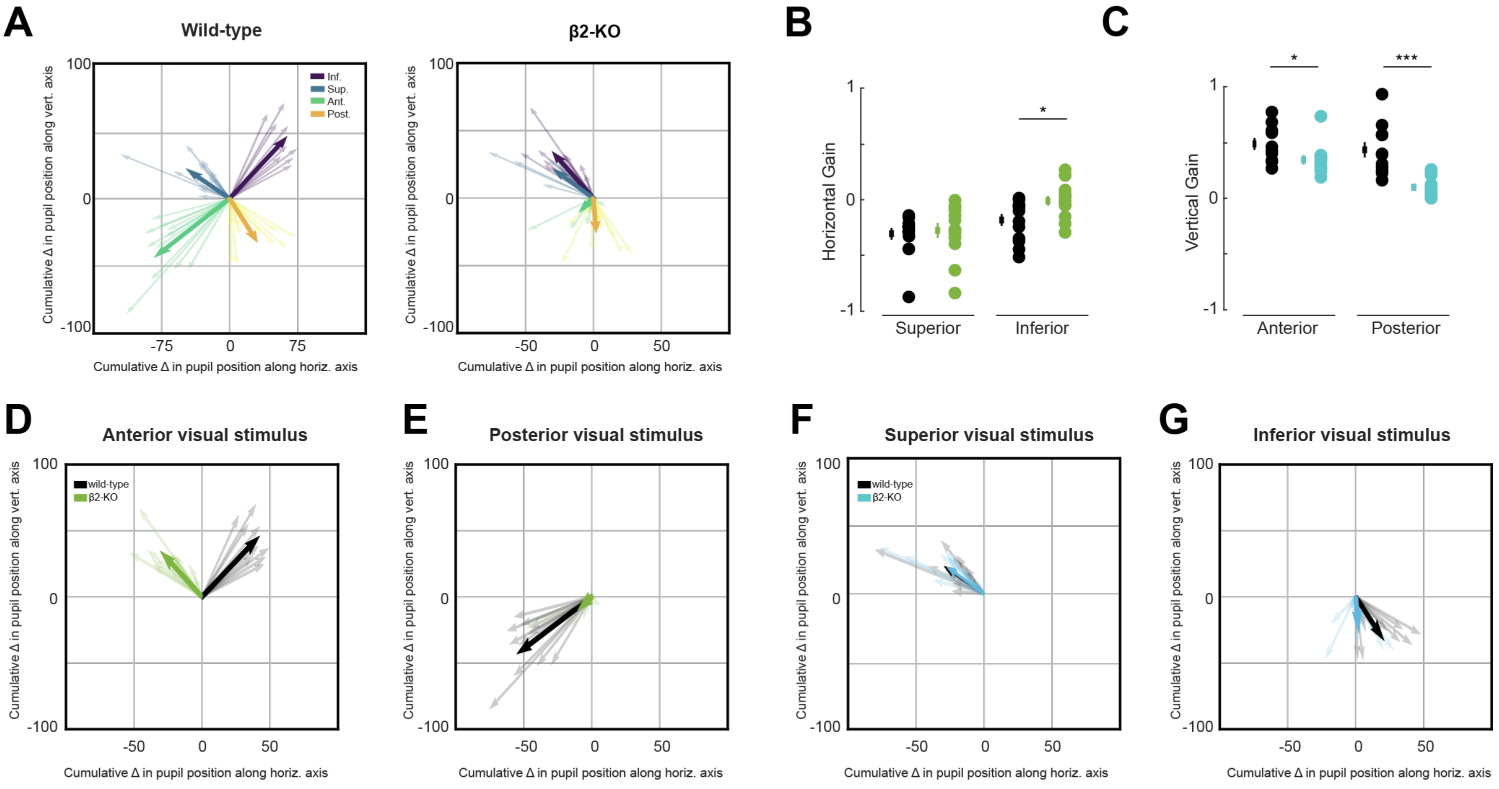

Figure S1: Optokinetic response to four cardinal directions in wild-type and β2-nAChR-KO mice. Related to Figure 1.

(A) Cumulative change in pupil position (degrees) during slow pursuit in wild-type (left) and β2-nAChR-KO (right) mice. Different colors indicate responses to various stimulus directions. Dark lines represent the average response, while light lines show individual trials. The length of each line reflects the total distance the pupil traveled during slow pursuit in that trial. Positive axis indicates posterior, negative x indicates anterior, positive y indicates superior, and negative y indicates inferior movement. *n* = 3 wild-type mice and 3 β2-nAChR-KO mice.

(B) Horizontal gain for superior and inferior motion in wild-type (black) and β2-nAChR-KO (green) mice. This measures the horizontal pupil movement in response to a vertical stimulus, where positive x indicates posterior movement and negative x indicates anterior movement. $*p < 0.05$; Unpaired t-test. *n* = 3 wild-type mice and 3 β2-nAChR-KO mice. Thick bars represent mean ± SE.

(C) Vertical gain for posterior and inferior motion in wild-type (black) and β2-nAChR-KO (blue) mice. This measures the vertical pupil movement in response to a horizontal stimulus. $*p < 0.05$; $***\ p < 0.001$; Unpaired t-test. *n* = 3 wild-type mice and 3 β2-nAChR-KO mice. Thick bars represent mean ± SE.

(D-G) Cumulative change in pupil position (degrees) during slow pursuit in wild-type and β2-nAChR-KO mice. Same as A but each graph corresponds to an individual motion direction: anterior (D), posterior (E), superior (F), and inferior (G).

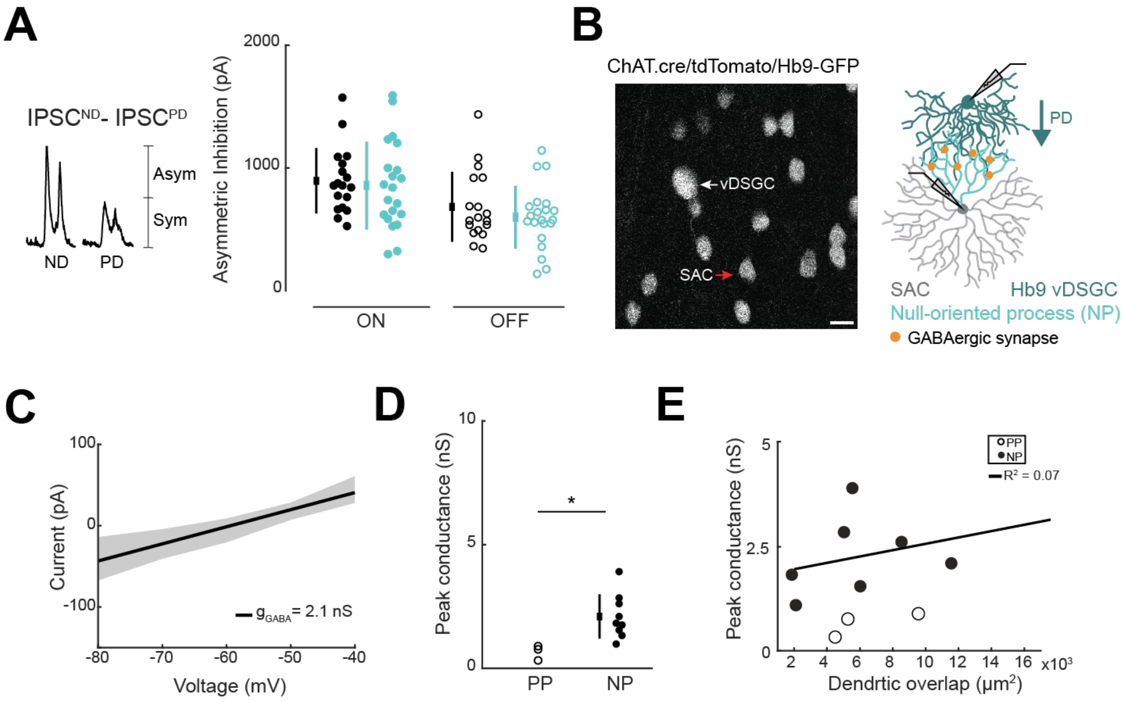

Figure S2: Ventral-preferring ON-OFF DSGCs receive comparatively weak GABAergic conductance from null-oriented SAC processes in wild-type and β2-nAChR-KO retinas. Related to Figure 3.

(A) Asymmetric inhibition of wild-type (black) and β2-nAChR-KO VDSGCs (green) for both the ON (filled) and OFF (open) responses. The asymmetric inhibitory component was calculated by subtracting the preferred direction IPSC from the null direction IPSC. $P > 0.05$; Unpaired t-test.

(B) Schematic depicting dual whole-cell recording between vDSGCs and SACs. Scale bar, 10µm.

(C) Average current-voltage relationship across all recorded null-oriented pairs for wild-type (black) and β 2-nAChR-KO (blue) retinas, where the slope of each line represents the average GABAergic conductance (gGABA), and the shaded regions represent the standard deviation.

(D) Peak GABAergic conductance from preferred- and null-oriented processes (PP, open circles; NP, filled circles) in wild-type (black) and null-oriented processes in β2-nAChR-KO retinas (blue). $n = 2$ preferred-oriented pairs and 7 null-oriented pairs across 4 wild-type retinas and 4 null-oriented pairs across 3 β2-nAChR-KO retinas. * $p < 0.05$; One-way ANOVA followed by Tukey-Kramer post hoc test. Error bars show mean +/- standard deviation.

(E) Peak GABAergic conductance as a function of dendritic overlap between vDSGC and SACs. Blue line represents correlation for β2-nAChR-KO null-oriented pairs (blue filled circles) where the correlation coefficient (R^2) equals 0.96. No correlation was found for null-oriented (black filled circles) or preferred-oriented (black open circles) pairs in wild-type retinas. Pairs with unclear images were excluded from convex hull analysis.

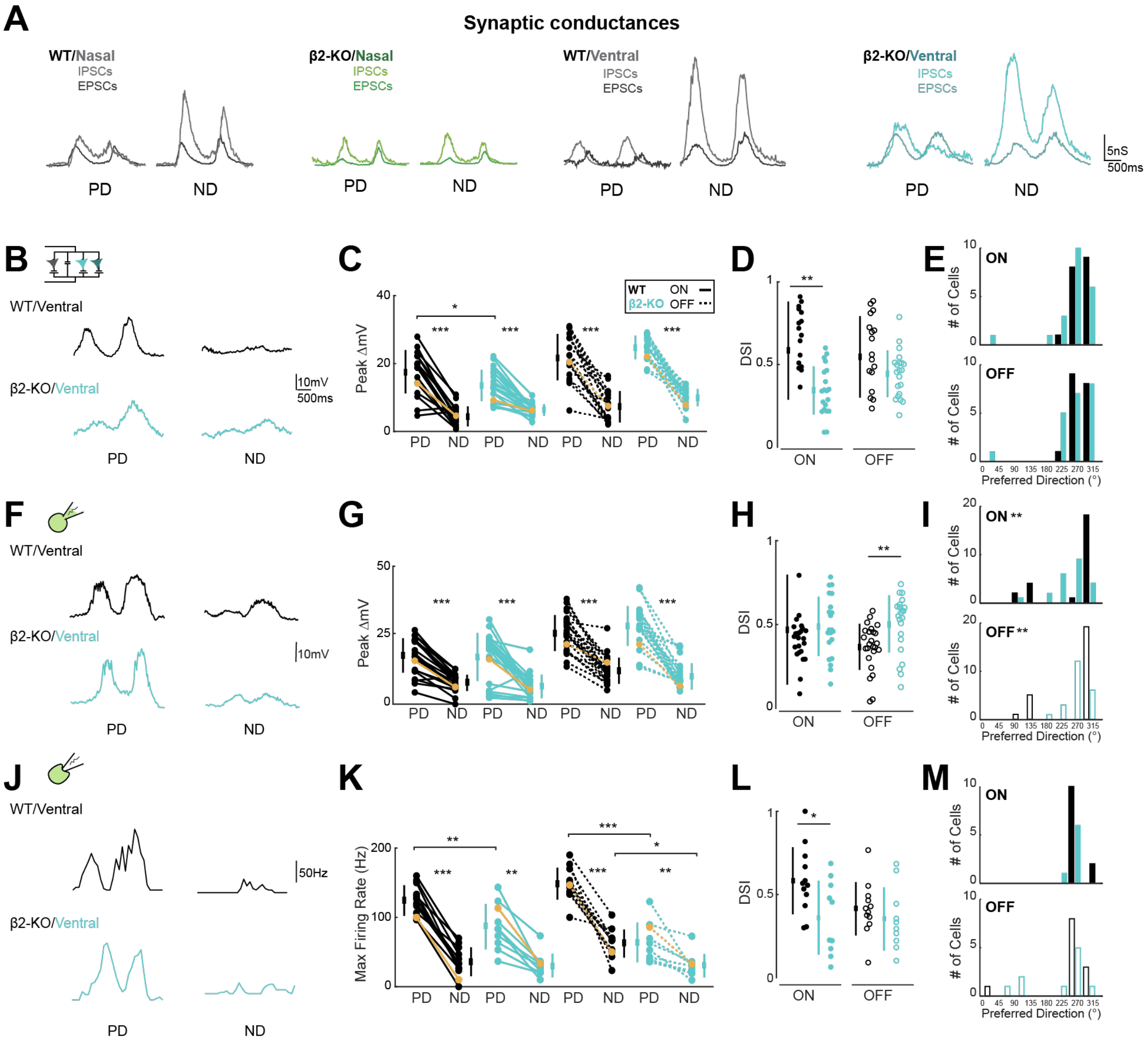

Figure S3: Ventral-preferring ON-OFF DSGCs maintain tuned subthreshold potentials and spiking activity in β2-nAChR-KO retinas despite reduction in excitatory inputs. Related to Figure 5.

Figure S3: Ventral-preferring ON-OFF DSGCs maintain tuned subthreshold potentials and spiking activity in β2-nAChR-KO retinas despite reduction in excitatory inputs. Related to Figure 5.

(A) Left: Example recorded excitatory and inhibitory conductances (EPSCs,darker; IPSCs,lighter) from example nDSGC from wild-type (black) and β2-nAChR-KO (green) for preferred (PD) and null (ND) directions. Right: Example recorded EPSCs (darker) and IPSCs (lighter) from example vDSGC in wild-type (black) and β2-nAChR-KO (blue) for the PD and ND.

(B) Example simulated subthreshold membrane potential from example vDSGC in wild-type (black) and β2-nAChR-KO (blue) retina in the PD and ND.

(C) Peak change in simulated subthreshold membrane potential for vDSGCs in wild-type (black) and β2-nAChR-KO (blue) for the ON (solid) and OFF (dashed) response. Yellow lines indicate example cells in B. n = 16 vDSGCs across 5 wild-type and 20 vDSGCs across 5 β2-nAChR-KO retinas. ** $p < 0.01$; *** $p<0.001$; ANOVA followed by Tukey-Kramer post hoc test. Error bars show mean +/- standard deviation.

(D) Direction selectivity index (DSI) of simulated subthreshold membrane potential in vDSGCs for ON (filled) and OFF (open) in wild-type (black) and β2-nAChR-KO (blue) retinas. Error bars show mean +/- standard deviation.

(E) Linear histograms representing the preferred direction of simulated subthreshold potential in wild-type (black) and β2-nAChR-KO (blue) vDSGCs for the ON (top) and OFF (bottom) responses. Ventral is 270 degrees. N.S; Levene's test for equality of variances.

(F) Example recorded subthreshold membrane potential from example vDSGC in wild-type (black) and β2-nAChR-KO (blue) retina in the PD and ND. Spikes were removed using a low pass filter or TTX bath application.

(G) Peak change in recorded subthreshold membrane potential for vDSGCs in wild-type (black) and β2-nAChR-KO (blue) retinas for the ON (sold) and OFF (dashed) response. TTX and spikes removed data were grouped together. Yellow lines indicate example cells in F. n = 22 vDSGCs across 6 wild-type and 21 vDSGCs across 5 β2-nAChR-KO retinas. *** $p < 0.001$; ANOVA followed by Tukey-Kramer post hoc test. Error bars show mean +/- standard deviation.

(H) Direction selectivity index (DSI) of recorded subthreshold membrane potential in vDSGCs for ON (filled) and OFF (open) in wild-type (black) and β2-nAChR-KO (blue) retinas.

(I) Linear histograms representing the preferred direction of recorded subthreshold potential in wild-type (black) and β2-nAChR-KO (blue) vDSGCs for the ON (top) and OFF (bottom) responses. ** $p < 0.01$; Levene's test for equality of variances.

(J) Average firing rates for example vDSGC in wild-type (black) and β2-nAChR-KO (blue) retina across 3 trials of PD and ND stimulation.

(K) Maximum firing rate for vDSGCs in wild-type (black) and β2-nAChR-KO (blue) retinas for the ON (sold) and OFF (dashed) responses. Yellow lines indicate example cells in J. n = 12 vDSGCs across 4 wild-type and 10 vDSGCs across 4 β2-nAChR-KO retinas. *$p < 0.05$; **$p <0.01$; *** $p < 0.001$; ANOVA followed by Tukey-Kramer post hoc test. Error bars show mean +/- standard deviation.

(L) Direction selectivity index of maximum firing rate in vDSGCs for ON (filled) and OFF (open) in wild-type (black) and β2-nAChR-KO (blue) retinas. * $p < 0.05$; Unpaired t-test. Error bars show mean +/- standard deviation.

(M) Linear histograms representing the preferred direction of spike output in wild-type (black) andβ2-nAChR-KO (blue) vDSGCs for the ON (top) and OFF (bottom) responses. $p > 0.05$; Levene's test for equality of variances.
